# Moisture-Controlled Electrolyte Engineering Enables Durable Calcium-Ion Batteries

**DOI:** 10.3390/mi17040390

**Published:** 2026-03-24

**Authors:** Yeon Jwoong Kim, Tejaswi Tanaji Salunkhe, Il Tae Kim

**Affiliations:** School of Chemical, Biological, and Battery Engineering, Gachon University, Seongnam-si 13120, Gyeonggi-do, Republic of Korea

**Keywords:** calcium-ion batteries, molecular sieve-assisted drying, electrolyte dehydration engineering, multivalent ion electrochemistry, acetonitrile

## Abstract

Calcium-ion batteries (CIBs) offer several advantages. CIBs are viable alternatives to lithium-based battery systems owing to the natural abundance, low cost, and high volumetric capacity of calcium. However, their development has been severely constrained by electrolyte instability and water sensitivity. We conducted a systematic examination of Ca(ClO_4_)_2_ and Ca(PF_6_)_2_ electrolytes, focusing on low-cost salt production, solvent selection, and stringent dehydration procedures. Acetonitrile (ACN) was the ideal solvent for high salt solubility and reversible Ca^2+^ electrochemistry, while carbonate solvents failed rapidly. We found that even a small amount of moisture in the electrolyte significantly affected the electrochemical performance. This study improved the dehydration process by using 3 Å molecular sieve (MS3A) and vacuum drying to reduce moisture to ppm levels, stabilizing the electrolyte. Prussian blue (PB) half cells exhibited reversible capacities of up to ≈95 mAh g^−1^, whereas PB-hard carbon full cells utilizing dried Ca(ClO_4_)_2_ showed stable cycling over 240 cycles with a Coulombic efficiency of ≈99% and capacity loss of only ≈17%. This study establishes a moisture-controlled electrolyte as a critical enabler for practical CIBs.

## 1. Introduction

**Scheme 1:**
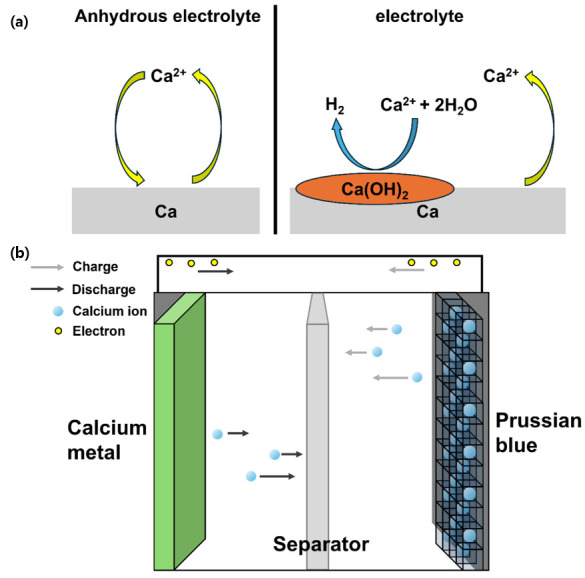
Schematic of (**a**) calcium metal in anhydrous electrolyte and (**b**) a calcium metal half-cell.

The rapid transition from gasoline engines to electric motors, as well as the widespread adoption of renewable energy sources, necessitates the development of next-generation rechargeable batteries that can overcome the constraints of existing lithium-ion battery technology [1,2,3]. Although lithium-ion batteries have achieved extraordinary commercial success, their finite natural abundance, unequal geopolitical distribution, growing costs, and intrinsic safety risks linked to dendritic lithium growth pose increasing hurdles to long-term sustainability [4,5]. These challenges have sparked a surge in interest in alternative charge carriers based on abundant material resources. Calcium-ion batteries (CIBs) are a promising option owing to their abundant natural supply, inexpensive cost, low redox potential (−2.87 V versus SHE), and high volumetric capacity (2073 mAh cm^−3^). Furthermore, the divalent nature of Ca^2+^ allows for the transfer of two electrons per ion, leading to high-energy-density storage with minimal material utilization [6]. In this context, the present study specifically focuses on nonaqueous CIBs, where electrolyte chemistry and moisture sensitivity play a decisive role in governing Ca^2+^ transport, interfacial stability, and electrochemical reversibility (Scheme 1a). In this context, the present study specifically focuses on nonaqueous CIBs, where electrolyte chemistry and moisture sensitivity play a decisive role in governing Ca^2+^ transport, interfacial stability, and electrochemical reversibility.

Despite the inherent advantages of calcium, fundamental electrolyte issues have significantly hampered the practical implementation of nonaqueous CIBs (Scheme 1b). Ca^2+^ has significant electrostatic interactions with solvent molecules and counter anions, resulting in slow ion transport, high desolvation barriers, and unstable interphases at the electrode–electrolyte interface [7,8]. Electrolyte chemistry plays a crucial role in the development of calcium-based energy storage systems, affecting not only the transport kinetics but also the electrode reversibility, cycle life, and rate capability. Ca(ClO_4_)_2_ and Ca(PF_6_)_2_ are two of the most extensively studied options among the reported calcium salts owing to their comparatively weak anion–cation interactions and possible compatibility with organic solvents [9,10,11]. However, both salts present significant and unique challenges [12]. Commercial Ca(ClO_4_)_2_ is usually produced as a highly hydrated salt with water molecules that are tightly coupled to Ca^2+^, which causes parasitic side reactions and severely impairs the electrochemical stability. Conversely, Ca(PF_6_)_2_ is inherently sensitive to moisture and susceptible to chemical and photochemical breakdown, producing reactive species that deteriorate the integrity of the electrodes and electrolyte [2,13]. Moreover, systematic investigations linking the synthesis, dehydration method, solvation behavior, and electrochemical performance are notably lacking, and few dependable, affordable synthesis techniques for high-purity Ca(PF_6_)_2_ have been reported.

The performance of the calcium electrolyte is significantly influenced by the type of solvent used. In addition to stabilizing the Ca^2+^ solvation shell, facilitating effective desolvation at the electrode interfaces and offering a sufficiently broad electrochemical stability window, the solvent must concurrently dissolve the calcium salt at realistic concentrations [14,15]. Compared with traditional carbonate solvents, acetonitrile (ACN) is shown to be a particularly advantageous solvent in this study because of its high dielectric constant, low viscosity, excellent solvating ability for Ca^2+^, and superior electrochemical stability. However, carbonate-based electrolyte systems, such as Ethylene Carbonate (EC) and Propylene Carbonate (PC), do not work well with calcium salts, leading to excessively high overpotentials and rapid cell failure. Crucially, we show that the electrochemical reversibility and salt solubility of ACN are highly sensitive to trace moisture, highlighting the crucial need for thorough dehydration. In nonaqueous CIBs systems, calcium electrochemistry is severely compromised by water contamination originating from hydrated salts, air exposure, or solvent impurities [12]. Even trace levels of water in nonaqueous electrolytes can induce salt decomposition, promote the formation of electronically insulating interphases, and severely suppress reversible Ca^2+^ transport. Therefore, the effective dehydration of both Ca(ClO_4_)_2_ and Ca(PF_6_)_2_ is essential. Two complementary dehydration techniques are thoroughly evaluated in this study: molecular sieve 3 Å (MS3A)-assisted dehydration, which selectively absorbs residual trace moisture from the salts, and extended vacuum drying, which removes structurally bound water. We demonstrate that MS3A treatment is particularly successful in reducing the water content to the ppm level, which stabilizes the electrolyte chemistry and greatly improves the electrochemical performance.

To assess the practical implications of the electrolyte chemistry and dehydration, we assemble Ca symmetric cells, Prussian blue (PB) half cells, and calcium-ion full cells with hard carbon (HC) anodes and PB cathodes. PB is used as a standard cathode because of its open framework and advantageous Ca^+^ transport pathways, whereas HC offers a stable and scalable anode platform. Our electrochemical investigation shows that thoroughly dehydrated Ca(ClO_4_)_2_ electrolytes enable highly reversible Ca^2+^ storage, resulting in long-term cycling stability of over 240 cycles and Coulombic efficiencies approaching 99%. Ca(PF_6_)_2_ electrolytes have greater high-rate capability but experience rapid capacity fading, indicating anion-dependent trade-offs between kinetics and stability. Overall, this study provides a complete and mechanistic understanding of calcium electrolyte chemistry. This study also explores salt synthesis, solvent selection, moisture control, and full-cell performance. This study establishes practical dehydration strategies and elucidates the structure–property–performance relationships of Ca(ClO_4_)_2_ and Ca(PF_6_)_2_ electrolytes, providing critical design principles for advancing CIBs as viable, low-cost, and sustainable energy-storage technologies.

## 2. Materials and Methodology

### 2.1. Material Synthesis

#### 2.1.1. Synthesis of Ca(PF_6_)_2_ Electrolyte Salt

Anhydrous calcium chloride (CaCl_2_) was obtained by vacuum-drying calcium chloride dihydrate (CaCl_2_·2H_2_O, 99–105%, Alfa AesarWard Hill, MA, USA) at 190 °C for 72 h. Subsequently, AgPF_6_ (2 mmol, 98%, Thermo Scientific, Waltham, MA, USA) was dissolved in 15 mL of anhydrous ACN (99.9%, Honeywell, Charlotte, NC, USA) under an inert atmosphere [16,17]. The resulting solution was then added dropwise to a suspension of CaCl_2_ (1.5 mmol) in 35 mL of anhydrous ACN with continuous magnetic stirring. The reaction mixture was stirred at room temperature for 24 h to ensure complete ion exchange. After completion, the mixture was filtered through a GF/C glass fiber filter to remove the precipitated AgCl by-product. The filtrate was concentrated using a rotary evaporator in an oil bath at 30–50 °C to remove most of the solvent, followed by vacuum (10 mbar) drying at room temperature overnight to eliminate residual solvent. The resulting Ca(PF_6_)_2_ powder was collected and stored in an argon-filled glove box (H_2_O and O_2_ < 0.1 ppm) to prevent hydrolysis.

(1) $$CaCl_{2(s)}+2AgPF_{6(l)}\to Ca{\left(PF_{6}\right)}_{2(l)}+2AgCl_{\left(s\right)}$$

#### 2.1.2. Synthesis of Anhydrous Ca(ClO_4_)_2_ Electrolyte Salt

CaCO_3_ (0.05 mol) (JUNSEI, Tokyo, Japan) was added to 45 mL of DI water, and the suspension was stirred while 0.10 mol of 70% HClO_4_ (SAMCHUN, Seoul, Republic of Korea) was introduced. The reaction mixture was stirred at ambient temperature for 2 h to ensure complete conversion of CaCO_3_ to calcium perchlorate and evolution of CO_2_. After stirring, the solvent was removed via vacuum filtering followed by heating at 140 °C until a crystalline hydrate (Ca(ClO_4_)_2_·xH_2_O) was obtained.

(2) $$CaCO_{3(s)}+2HClO_{4(l)}\to Ca{\left(ClO_{4}\right)}_{2(l)}+H_{2}O_{\left(l\right)}+CO_{2(g)}$$

**Figure 1:**
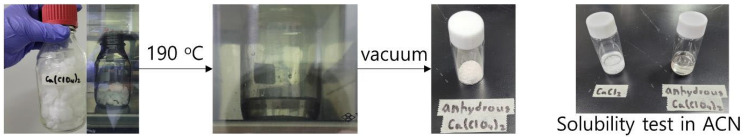
Process of conversion of hydrated salt Ca(ClO_4_)_2_ to the anhydrous salt and solubility test.

The hydrated salt was converted to the anhydrous salt by prolonged thermal desiccation under reduced pressure (Figure 1). The partially dried material was placed in a vacuum oven at 190 °C and held under vacuum for 7 days. During the initial stages of drying, the material appeared liquid due to retained water; therefore, the drying cycle was accelerated by periodically backfilling the oven with dry argon to reduce the partial pressure of H_2_O and then reapplying the vacuum. This argon refill/vacuum cycle was repeated until the sample solidified, and no further mass loss was observed. The final anhydrous Ca(ClO_4_)_2_ was handled and stored under an inert atmosphere (Ar) in a dry glove box (H_2_O and O_2_ < 0.1 ppm) to avoid rehydration.

#### 2.1.3. Synthesis of Prussian Blue Cathode Material

PB was synthesized using a simple aqueous co-precipitation method. Sodium ferrocyanide decahydrate (Na_4_Fe(CN)_6_·10H_2_O, 98%, Sigma-Aldrich, Burlington, MA, USA, 0.01 mol) was dissolved in 40 mL of deionized (DI) water, while anhydrous iron(III) chloride (FeCl_3_, DAEJUNG, Siheung-si, Gyeonggi-do, Republic of Korea, 0.01 mol) was separately dissolved in 10 mL of DI water. The FeCl_3_ solution was added dropwise to the sodium ferrocyanide solution under continuous magnetic stirring at 60 °C, and the reaction was maintained for 2 h to ensure complete precipitation [18,19]. To eliminate residual ions and unreacted precursors, the blue precipitate was separated by centrifugation (10,000 rpm, 15 min) and rinsed three times each with ethanol and DI water. The final PB powder was obtained by vacuum-drying the purified product at 90 °C for 6 h [20].

### 2.2. Material Preparation

#### 2.2.1. Activation of Molecular Sieve 3Å (MS3A)

Molecular sieves (MS3A, 3 Å, 1–2 mm beads, Thermo Scientific, Waltham, MA, USA) were activated prior to use by heating at 330 °C overnight in a furnace [21]. If residual moisture remained, additional drying was performed until the sieves were completely dry. The activated MS3A was then transferred to a glass bottle, sealed immediately after cooling, and wrapped with parafilm to prevent moisture uptake. The activated MS3A was then used to remove moisture from the solvent and electrolyte solutions. After reaching their full moisture absorption capacity, spent MS3A beads were regenerated by re-drying at 330 °C and reused multiple times [22].

#### 2.2.2. Anhydrous Solvent Preparation

Activated MS3A was mixed with the solvent at a 1:4 volume ratio in a glass bottle. The mixture was shaken for 30 min, sealed with parafilm, and kept at room temperature for three days to allow complete moisture absorption. After the MS3A had settled, the solvent was collected using a pipette and filtered through GF/C filter paper. All solvent drying and handling procedures were conducted inside a dry argon-filled glove box, and the purified anhydrous solvent was stored under the same conditions until use.

#### 2.2.3. Ca Metal Electrode Preparation

Calcium metal granules (99%, ACROS Organics, Geel, Belgium) were stored in an argon glove box and handled while immersed in mineral oil to prevent oxidation in air. The Ca pieces were placed between stainless-steel molds, wetted with mineral oil, and compressed using a hydraulic press at 20–35 MPa to form metal foils with a thickness of 700–900 μm [23]. The pressed foil was punched into a circular electrode (10 mm diameter) and immediately transferred to a glove box. The foils were rinsed with hexane to remove residual mineral oil from the surface and were subsequently polished using a sanding tool until a uniform, bright silver-white surface was obtained.

#### 2.2.4. Calcium Metal Symmetric Cell and PB-HC Full Cell

To create the calcium metal symmetric cell and the calcium metal full cell, CR2032 coin cells were assembled in an Ar glove box. A glass-fiber filter (GF/A) was used as the separator. Calcium metal symmetric cells were prepared using Ca(PF_6_)_2_ electrolytes in various solvents (EC-PC, PC, PC-ACN, PC-monoglyme, ACN-monoglyme, ACN-triglyme) at a concentration of 0.5 M. Symmetric cells were prepared using Ca(ClO_4_)_2_ electrolytes in various solvents (EC-PC, PC, PC-ACN, and ACN) at a concentration of 1 M.

PB was used as the active cathode material. The cathode electrode was made by vacuum-drying a slurry containing PB, poly(vinylidene fluoride) (PVDF, Mw = 534,000, Sigma Aldrich, Burlington, MA, USA), and carbon black (super P, CB, 99.5%, Alfa Aesar, Ward Hill, MA, USA) in *N*-methyl-2-pyrolidone (NMP, 99.5%, Sigma Aldrich) and casting the slurry in SS316 foil (0.01 mm × 100 mm × 1000 mm, T × W × L, MTI, Seoul, Republic of Korea). The slurry was prepared by placing it in NMP at a PB:PVDF:carbon black ratio of 8:1:1 (wt ratio) while stirring. Ca(ClO_4_)_2_ (1 M) and Ca(PF_6_)_2_ (0.5 M) in ACN were used as electrolytes for full cell. As well as it has been evaluated in half cell with commercial Ca(ClO_4_)_2_ salt, commercial Ca(ClO_4_)_2_ salt + MS3A, synthesized Ca(ClO_4_)_2_ salt, synthesized Ca(ClO_4_)_2_ salt + MS3A, synthesized Ca(PF_6_)_2_ salt, and synthesized Ca(PF_6_)_2_ + MS3A.

The anodes were fabricated using HC as the active material with an HC:PVDF:Super P weight ratio of 70:15:15. The components were first homogenized by dry mixing to obtain a uniform powder blend, followed by the addition of NMP to form a slurry, which was stirred for 24 h. The resulting slurry was uniformly cast onto copper foil to achieve a final coating thickness of approximately 15 µm. The coated electrodes were then dried under vacuum at 70 °C for at least 12 h to remove residual solvent and enhance the mechanical stability. After drying, the electrodes were cut to the desired size, weighed to determine the active material loading, and subsequently transferred to an argon-filled glove box for cell assembly full cell. No pre-calcification step was employed in this study; instead, Ca^2+^ ions were supplied exclusively by the electrolyte salt and reversibly shuttled between electrodes during electrochemical cycling.

### 2.3. Material Characterization

The morphology and elemental composition of the synthesized Ca(PF_6_)_2_ electrolyte salt were examined using scanning electron microscopy (SEM; Hitachi S4700, Tokyo, Japan) coupled with energy-dispersive X-ray spectroscopy (EDX; Hitachi S4700) to confirm the particle structure and purity. The moisture contents of the solvents and electrolytes were quantified using Karl Fischer titration (KF; Compact C10S, Mettler Toledo, Greifensee, Switzerland). To determine the amount of water bound to the electrolyte salt in the form of hydrates, the salt was dissolved in solvent at a 5:95 (wt%) ratio, and titration was performed using HYDRANAL^TM^ Coulomat AG (Honeywell, Seelze, Germany) as the electrolyte solution in the KF cell. SEM analysis was conducted to examine the morphology of the PB particles, and EDX measurements were performed to confirm their elemental composition. The Phase purity of the PB has been measured by X-ray diffraction (XRD) by (XRD, Rigaku SmartLab, Tokyo, Japan).

### 2.4. Electrochemical Measurements

Galvanostatic charge–discharge measurements of each coin cell were performed using a battery cycler system (WBCS300, WonATech, Seoul, Republic of Korea). Electrolytes included 0.5 M Ca(PF_6_)_2_, synthesized from AgPF_6_, in ACN and mixed solvents, as well as 1 M Ca(ClO_4_)_2_ in ACN and ACN–PC mixed solvents. Symmetric Ca metal cells were tested at a current density of 0.1 A cm^−2^ with varying time intervals to evaluate the reversibility and staAbility of the calcium deposition and stripping. Half-cell measurements were conducted using PB as the cathode with 1 M Ca(ClO_4_)_2_ in ACN at a current density of 0.03 A g^−1^ within a voltage window of 0–3 V. Comparative evaluations were carried out using (i) undried commercial Ca(ClO_4_)_2_ electrolyte, (ii) commercial salt dried with MS3A, (iii) synthesized Ca(ClO_4_)_2_ electrolyte dried under vacuum, and (iv) synthesized Ca(ClO_4_)_2_ salt dried under both vacuum and MS3A. Additional tests were performed with 1 M Ca(PF_6_)_2_ and Ca(PF_6_)_2_ + MS3A electrolytes in ACN. The full-cell electrochemical performance was examined using an HC anode and a PB cathode (HC–PB) at a current density of 0.1 A g^−1^ within a voltage range of 0–2 V. Galvanostatic charge–discharge cycling and rate-capability tests were conducted using 1 M Ca(ClO_4_)_2_ in ACN and 0.5 M Ca(PF_6_)_2_ in ACN, both subjected to drying with MS3A.

## 3. Results and Discussion

**Figure 2:**
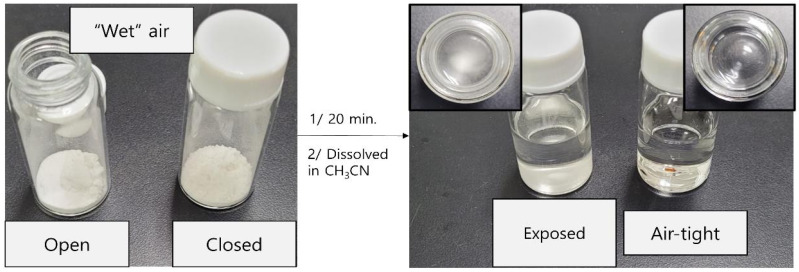
Solubility test of Ca(PF_6_)_2_ in acetonitrile: (**left**) exposed to ambient air and (**right**) stored in an airtight environment.

The obtained Ca(PF_6_)_2_ salt was a white crystalline solid that was extremely sensitive to moisture. Because of the humidity, the salt quickly decomposed after being exposed to the surrounding air for a few minutes. Ca(PF_6_)_2_ is extremely soluble in ACN (approximately 71 g per 100 mL) and highly hygroscopic; thus, controlling moisture during handling is crucial. Ca(PF_6_)_2_ was evaluated in two circumstances to assess the impact of moisture on its solubility: (i) exposed to ambient air and (ii) stored in an airtight environment. Figure 2 shows that the airtight sample readily dissolved to create a clear solution, whereas the air-exposed (“wet”) Ca(PF_6_)_2_ became entirely insoluble in ACN. These results show that even trace amounts of absorbed water considerably impede the breakdown, which poses a significant obstacle to the synthesis of electrolytes.

**Figure 3:**
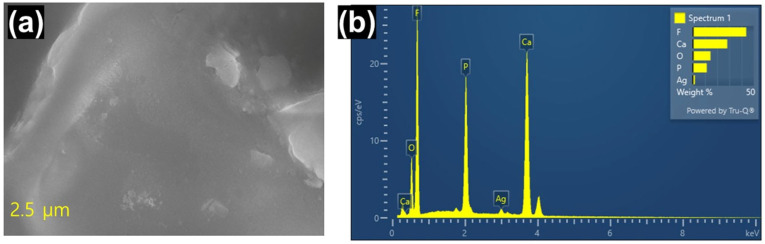
(**a**) SEM image of the synthesized Ca(PF_6_)_2_ and (**b**) EDX spectrum.

As shown in Figure 3, SEM-EDX was used to analyze the morphology and elemental content of the synthesized Ca(PF_6_)_2_ salt. Because Ca is highly sensitive to moisture, the SEM image in Figure 3a does not show a well-defined morphology or crystal structure. Partial hydrolysis was caused by brief exposure to ambient air during sample transfer, which is consistent with the increased oxygen signal observed in the EDX spectrum (Figure 3b and Table 1). Minor anion degradation is indicated by the P:F atomic ratio (1:6.21), which differs significantly from the ideal PF_6_^−^ stoichiometry (1:6) [24,25]. Additionally, the predicted value of 1:2:12 for Ca(PF_6_)_2_ is much higher than the Ca:P:F molar ratio reported via EDX (1:0.53:3.27), indicating a considerable loss of PF_6_^−^ species. This breakdown is likely caused by the inherent reactivity of PF_6_^−^ with trace moisture, wherein progressive hydrolysis yields HF and PF_5_, the latter of which is volatile under ambient conditions and causes detectable anion depletion [13,26,27]. These results unequivocally show that moisture-induced decomposition occurred during ex situ sample handling, even when the synthesis was performed inside a dry glove box. The detected Ag signal originates from residual AgPF_4_, which is not eliminated during filtration because of its high solubility in ACN. The product purity is expected to be approximately 97.7% based on the Ca:Ag molar ratio (1:0.023) and assuming minimal PF_6_^−^ breakdown. The carbon tape and SEM sample substrate were the sources of the Si and C contributions.

**Figure 4:**
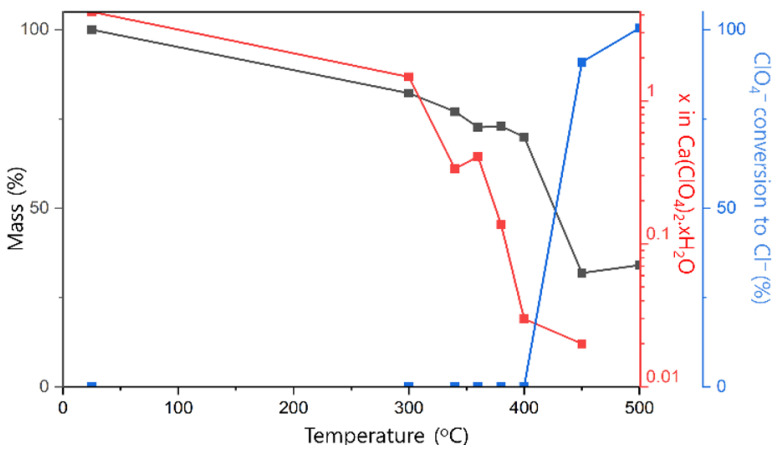
Thermal analysis of Ca(ClO_4_)_2_∙4H_2_O; the annealed sample was analyzed for x (Karl-Fischer titration, Mettler Toledo C10, Hydranal Coulomat AG, Jaipur, India).

Calcium perchlorate exhibits pronounced hygroscopicity, and the commercial Ca(ClO_4_)_2_ salt exists as a tetrahydrate (Ca(ClO_4_)_2_·4H_2_O). Consequently, rigorous dehydration is essential before electrolyte formulation. To identify an optimal dehydration temperature, thermogravimetric analysis (Figure 4) was performed on Ca(ClO_4_)_2_·4H_2_O samples annealed between 300 and 500 °C. Progressive mass loss from 300 to 400 °C corresponds to stepwise removal of coordinated water, while the ClO_4_^−^ anion remains stable throughout this temperature range. However, annealing at ≥450 °C induces significant ClO_4_^−^ decomposition, evidenced by conversion to CaCl_2_ upon dissolution of the annealed product in ACN. These results indicate that ≈390 °C represents the upper limit for thermal dehydration without inducing perchlorate degradation. In the subsequent experiment, it was found that the vacuum drying result at 190 °C was similar to the result of the above experiment, and subsequent experiments were conducted using vacuum drying.

The Karl Fischer titration results (Table 2) clearly demonstrate the progressive removal of moisture from Ca(ClO_4_)_2_ through sequential dehydration treatments. Commercial Ca(ClO_4_)_2_ contains a high water content of 11,281 ppm (≈1.12 wt%), corresponding to 4.1 mol of bound water per mole of salt. Treatment with MS3A alone reduces the moisture level to 4560 ppm, indicating partial removal of free and weakly bound water. In contrast, Synthesized Ca(ClO_4_)_2_ prolonged vacuum drying is substantially, decreasing the water content to 802.3 ppm. Notably, combining vacuum drying with subsequent MS3A-assisted dehydration further suppresses the residual moisture to 11.2 ppm for Synthesized Ca(ClO_4_)_2_, approaching the intrinsic moisture level of dry acetonitrile. These results demonstrate that MS3A is particularly effective at scavenging trace residual water once the bulk and coordinated hydration water has been removed by vacuum drying, enabling near-complete dehydration of the electrolyte salt.

These results demonstrate that MS3A is most effective when applied after moisture has been reduced to the sub-0.1 wt% range, which is consistent with the limited adsorption capacity of the desiccant. Combining the TGA and KF titration results confirmed that Ca(ClO_4_)_2_ was stably dehydrated without pyrolysis of anions through heat treatment near 390 °C or vacuum drying for a long duration. To remove the remaining trace-level moisture, an optional MS3A drying should be performed in parallel. This anhydrous Ca(ClO_4_)_2_ electrolyte was used in all subsequent Ca(ClO_4_)_2_ coin cells.

**Figure 5:**
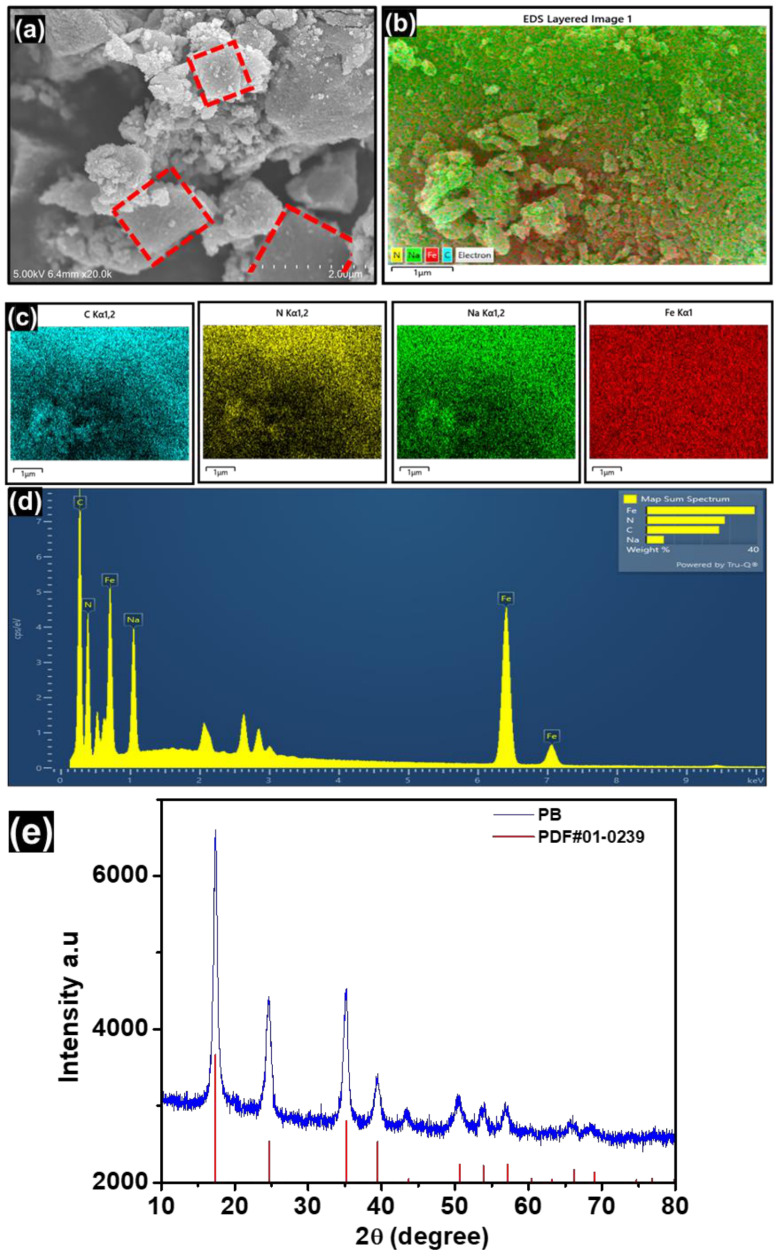
(**a**) SEM image of the synthesized PB, (**b**,**c**) EDX of elements, (**d**) EDX spectrum and (**e**) XRD pattern of hydrous Prussian Blue (Fe_4_[Fe(CN)_6_]_3_·xH_2_O).

The morphological and compositional properties of the synthesized PB were investigated using SEM and EDX, as illustrated in Figure 5. The SEM image (Figure 5a) illustrates that the PB particles have a highly agglomerated but distinguishable cubic morphology, composed of several interparticle gaps and irregularly shaped microcrystallites with rough surfaces. Apparently hierarchical architecture is advantageous for boosting effective electrochemical kinetics by shortening ion-diffusion paths and facilitating electrolyte penetration. The uniform spatial distribution of Na, Fe, C, and N throughout the entirety of the particle domain has been further demonstrated by elemental mapping (Figure 5b,c), suggesting that a uniform Prussian blue framework was successfully formed without any noticeable phase segregation. The chemical purity of the developed material is confirmed by the accompanying EDS spectrum (Figure 5d, which displays clear and strong signals of Na, Fe, C, and N with no discernible impurity elements. Reversible Ca^2+^ storage and sustained electrochemical performance in CIBs systems depend on favorable electronic connection and ion-accessible routes, which are suggested by the homogeneous elemental distribution and well-developed microstructure. The crystallographic phase of the Prussian Blue (PB) cathode material was examined by X-ray diffraction (XRD). As shown in Figure 5e, the diffraction pattern matches well with PDF card No. 01-0239, corresponding to low-hydrous Prussian Blue (Fe_4_[Fe(CN)_6_]_3_·xH_2_O) with a face-centered cubic structure (space group Fm-3m). The characteristic reflections at 2θ ≈ 17.4° (200), 24.6° (220), 35.1° (400), and 50.4° (440) confirm the formation of a structurally intact PB framework without detectable impurity phases. No reflections associated with dehydrated Prussian White or secondary iron cyanide phases were observed.

Given the hydrous nature of PB, the potential contribution of coordinated water to the electrolyte moisture content was quantitatively evaluated using Karl Fischer (KF) titration. PB electrodes were prepared following the same protocol used for electrochemical testing, including vacuum drying at 70 °C. A mixture of PB and acetonitrile (ACN) at a 5:95 weight ratio was sonicated, and the dissolved moisture was measured to be 2177 ppm based on 0.5 g of PB. When normalized to the actual cell configuration—an electrode loading of 1.131 mg PB and an electrolyte volume of 150 μL ACN (117.9 mg)—the PB-derived moisture contribution corresponds to only ~20.9 ppm. This value is significantly lower than the moisture levels originating from insufficiently dehydrated calcium salts and falls within the ppm regime shown to have a negligible influence on electrochemical performance. These results confirm that, although PB is structurally hydrous, its contribution to electrolyte moisture under practical cell conditions is minimal and does not affect the conclusions of this study.

**Figure 6:**
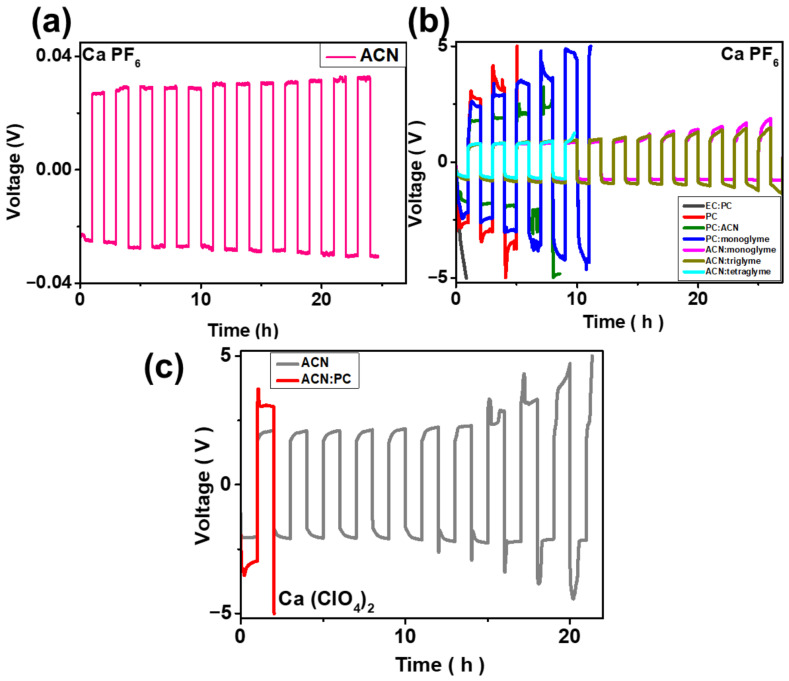
Voltage profiles of Ca metal symmetric cells using 0.5 M Ca(PF_6_)_2_ electrolytes synthesized from AgPF_6_ in (**a**) ACN and (**b**) mixed solvents, and 1 M Ca(ClO_4_)_2_ electrolyte in ACN and ACN–PC mixed solvents. (**c**) 1 M Ca(ClO_4_)_2_ electrolyte in in ACN and ACN–PC mixed solvents. All measurements were conducted at a current density of 0.1 mA cm^−2^.

Symmetric Ca–Ca cells were assembled to quantitatively evaluate the plating/stripping overpotential of calcium metal in well-defined electrolyte environments. All of the Ca(PF_6_)_2_ electrolytes were prepared at a concentration of 0.5 M, and mixed-solvent systems employed binary solvents at a 1:1 volume ratio. When 0.5 M Ca(PF_6_)_2_ (synthesized via metathesis from AgPF_6_) was dissolved in ACN, the Ca symmetric cell exhibited an exceptionally low overpotential of approximately 30 mV, indicative of highly facile Ca^2+^ desolvation and interfacial charge transfer kinetics (Figure 6a). Across many ACN-containing formulations, the Ca metal anode displayed remarkable reversibility during repeated plating and stripping. In contrast, electrolytes based on conventional carbonate solvents, specifically EC:PC mixtures commonly employed in Li- and Na-ion systems [17,28], failed to sustain even a single plating cycle on the Ca metal, underscoring their intrinsic incompatibility. Other PC-rich solvent systems also exhibited rapid cell failure, typically within fewer than six cycles. The initial interfacial overpotential further reflects these solvent-dependent behaviors: ACN-based electrolytes showed an initial overpotential of ≈0.6 V, whereas PC-only and ACN:PC (1:1 *v*/*v*) systems exhibited significantly higher values of ~3.13 V and 1.8 V, respectively (Figure 6b). To assess the salt dependence of these trends, symmetric cells employing 1 M Ca(ClO_4_)_2_ were also evaluated. It should be noted that the “initial overpotential” refers to the first-cycle transient polarization dominated by interfacial activation and Ca^2+^ desolvation barriers, whereas the voltage plateaus in Figure 6a,b correspond to the stabilized steady-state plating/stripping overpotential; therefore, these values are not expected to coincide quantitatively, particularly in kinetically hindered PC-containing electrolytes. The overall electrochemical behavior was qualitatively similar to that of Ca(PF_6_)_2_. In the PC:ACN (1:1 *v*/*v*) system, Ca(ClO_4_)_2_ produced an initial overpotential of ≈3 V, which was substantially higher than the >2 V observed for the ACN-only formulation, thus highlighting the strong influence of solvent identity on interfacial Ca deposition/desolvation barriers. Notably, the ACN-based Ca(ClO_4_)_2_ electrolyte sustained stable cycling for up to six cycles, whereas PC-containing systems again failed prematurely (Figure 6c).

**Figure 7:**
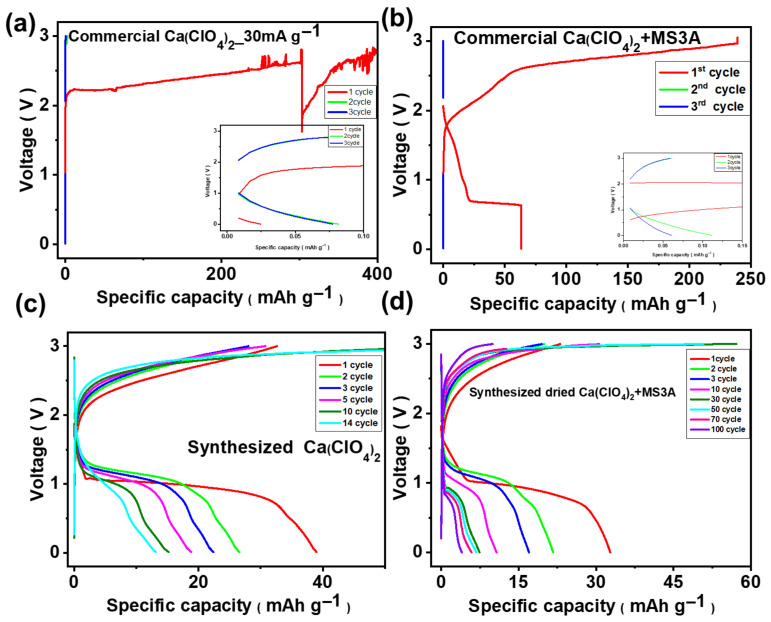
Charge–discharge voltage profiles of cells comprising a calcium metal anode and PB cathode using 1 M Ca(ClO_4_)_2_ in ACN at a current density of 0.03 A g^−1^: (**a**) commercial Ca(ClO_4_)_2_ salt electrolyte (undried), (**b**) commercial Ca(ClO_4_)_2_ salt dried with MS3A, (**c**) synthesized Ca(ClO_4_)_2_ salt electrolyte dried under vacuum, and (**d**) synthesized Ca(ClO_4_)_2_ salt electrolyte dried under vacuum and MS3A.

The electrochemical reversibility of calcium metal was first validated using Ca‖Ca symmetric cells. Based on the superior cycling stability observed in ACN, calcium metal batteries (CMBs) were assembled using PB as the cathode (cast on SS316 current collectors) and GF/A separators. For cells employing commercial Ca(ClO_4_)_2_ in ACN, the electrochemical performance was extremely poor. As shown in Figure 7a, the untreated electrolyte delivered only ≈0.08 mAh g^−1^ during the initial discharge, and no meaningful charge/discharge reversibility could be obtained, indicating that the intrinsic moisture content critically suppressed the Ca-ion electrochemistry. When the commercial salt was partially dehydrated using MS3A molecular sieves, the first discharge exhibited a more typical voltage profile; however, the subsequent charge/discharge immediately failed, with a capacity of only ≈0.11 mAh g^−1^ (Figure 7b), demonstrating that minimal drying is insufficient to stabilize the electrode–electrolyte interface. In contrast, the synthesized Ca(ClO_4_)_2_ salt, rigorously vacuum dried for one week, enabled reversible Ca-ion storage for up to 14 cycles (Figure 7c). Nevertheless, the cell exhibited a rapid capacity decay, with the discharge capacity falling to ≈20 mAh g^−1^ by the fifth cycle (approximately half of the first-cycle value), highlighting the remaining instability associated with Ca(ClO_4_)_2_. Remarkably, when the synthesized Ca(ClO_4_)_2_ was further conditioned with MS3A, the electrochemical performance improved dramatically, yielding charge–discharge profiles for up to 100 cycles (Figure 7d). Although partial overlap of the voltage profiles is observed during the initial cycles, a gradual decrease in capacity is evident within the first 10 cycles, indicating progressive electrochemical stabilization rather than complete voltage-profile retention.

**Figure 8:**
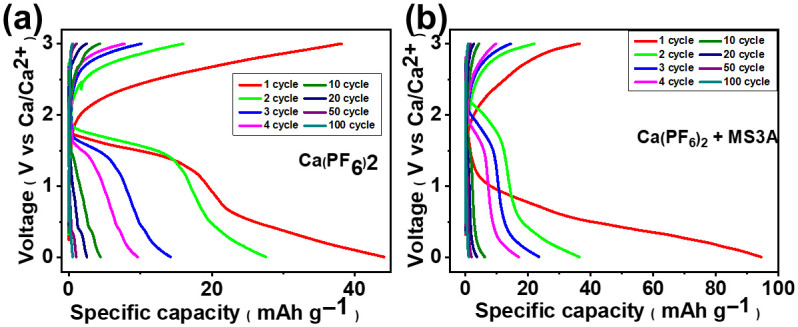
Charge/discharge voltage profiles of the half-cell with the PB cathode at a current density of 30 mA g^−1^ and a voltage window 0–3 V: (**a**) Ca(PF_6_)_2_, (**b**) Ca(PF_6_)_2_ + MS3A electrolyte in ACN solvent.

With the Ca(PF_6_)_2_-based electrolyte, the charging and discharging behavior was confirmed 100 times regardless of the additional MS3A drying process. However, compared with the Ca(ClO_4_)_2_-based electrolyte, the capacity decreased rapidly owing to the low stability of the electrolyte. The initial discharge capacity reached ≈44 mAh g^−1^, which decreased to 10 mAh g^−1^ by the fourth cycle and ≈5 mAh g^−1^ by the tenth cycle (Figure 8a). However, after MS3A-assisted drying, the Ca(PF_6_)_2_ electrolyte delivered a significantly higher first-cycle discharge capacity of 94.5 mAh g^−1^, which was the highest among all of the evaluated electrolytes (Figure 8b). Although this value rapidly decreased to ≈38 mAh g^−1^ in the second cycle and continued to decrease thereafter, the capacity retention remained superior to that of cells assembled with Ca(ClO_4_)_2_ electrolytes over the same number of cycles [29,30,31]. This highlights not only the intrinsic advantages of the PF_6_^−^ anion in ACN but also the essential role of rigorous dehydration in achieving kinetically favorable Ca-ion insertion and extraction in PB cathodes.

**Figure 9:**
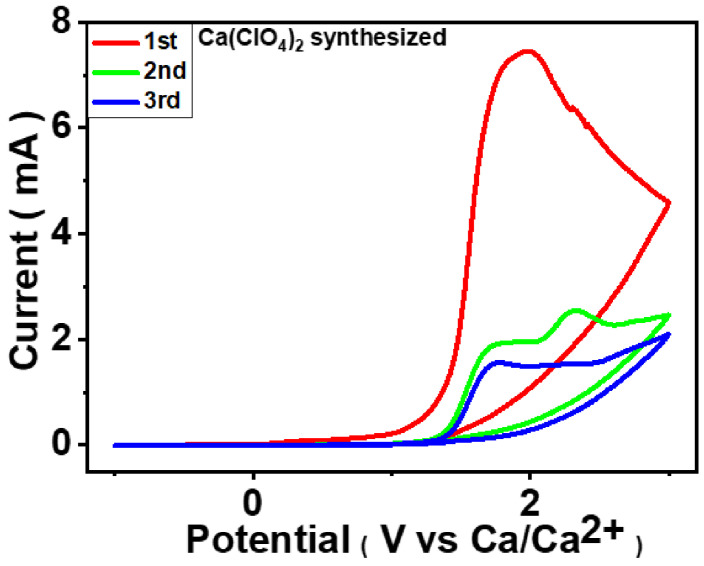
Cycling voltammetry of the 1 M synthesized Ca(ClO_4_)_2_ in ACN electrolyte dried under vacuum and MS3A.

The cyclic voltammetry (CV) profiles of the Prussian Blue (PB) cathode paired with a Ca metal anode elucidate the interfacial and bulk electrochemical processes governing Ca^2+^ storage (Figure 9). During the first anodic scan, a broad and pronounced oxidation feature appears at ~1.4–1.6 V, which is largely irreversible, as no corresponding cathodic peak is observed. This behavior is attributed to initial interfacial activation, including electrolyte decomposition and the formation of a passivating Cathode Electrolyte Interphase (CEI) on the PB surface, driven by the strong solvation and sluggish desolvation kinetics of Ca^2+^ ions. In subsequent cycles, the anodic current is significantly reduced, and the CV curves largely overlap, indicating suppression of parasitic reactions and stabilization of the interphase. The remaining electrochemical response arises predominantly from reversible Ca^2+^ insertion/extraction within the open PB framework. The absence of sharp redox peaks and the sloping current response reflect diffusion-limited, solid-solution–type Ca^2+^ storage kinetics associated with divalent ion transport. The enhanced overlap between later cycles confirms improved reversibility and reduced interfacial resistance, consistent with the disappearance of the first-cycle voltage plateau in galvanostatic profiles. Overall, the CV results demonstrate that stable CEI formation is essential for enabling reversible Ca^2+^ storage in PB cathodes.

**Figure 10:**
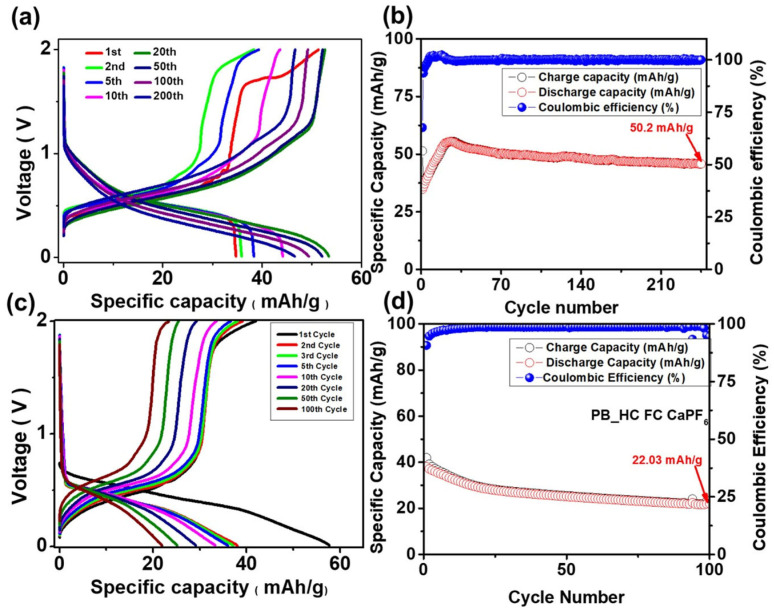
(**a**) Charge–discharge profiles and (**b**) cycling performance of the HC–PB full cell employing the synthesized 1 M Ca(ClO_4_)_2_ electrolyte in ACN dried under vacuum and MS3A, tested over a voltage window of 0–2 V at a current density of 0.1 A g^−1^; (**c**) charge–discharge profile and (**d**) cycle performance of the PB-HC full cell with Ca(PF_6_)_2_ electrolyte over the voltage window of 0–2 V at 0.1 A/g.

A full cell incorporating the rigorously dehydrated 1.0 M Ca(ClO_4_)_2_ electrolyte in ACN dried under vacuum and further conditioned with MS3A was assembled using PB as the cathode and HC cast on Cu foil as the anode. HC was selected instead of metallic calcium to mitigate the well-known challenges associated with Ca plating/stripping kinetics and to evaluate the Ca-ion intercalation behavior in a practical full-cell configuration. Galvanostatic cycling was performed over a voltage window of 0–2 V, yielding an initial discharge capacity of 55.5 mAh g^−1^ (Figure 10a). Interestingly, the discharge capacity gradually increased from cycle 1 to approximately cycle 20. This progressive activation is a characteristic feature frequently observed in Ca(ClO_4_)_2_-based electrolytes and has been previously reported in PB|Ca full cells employing the same salt system [32]. This phenomenon is attributed to several synergistic effects: (i) the slow formation and stabilization of a Ca-conductive interphase on the HC surface, (ii) enhanced wetting and ion transport through microporous regions of PB and HC as the electrolyte further equilibrates, and (iii) gradual removal of trace residual water through electrochemical consumption during early cycling, resulting in improved Ca^2+^ desolvation kinetics. Together, these interfacial and ionic-transport improvements manifest as an apparent “capacity activation” in the early cycles. Following activation, the discharge capacity reached a maximum near cycle 25; thereafter, gradual capacity decay was observed. Even at 240 cycles, which is well beyond conventional benchmarks for Ca-ion systems, the full cell retained a capacity of 45.8 mAh g^−1^, corresponding to a modest 17.4% reduction relative to the peak value. Throughout cycling, the Coulombic efficiency remained exceptionally stable at approximately 99% (Figure 10b), indicating highly reversible Ca-ion intercalation/deintercalation and the absence of significant parasitic reactions. Previous reports have established that Ca(ClO_4_)_2_ in ACN exhibits a wide electrochemical stability window (ESW > 3 V) [32] that is considerably larger than the cycling window employed here. Therefore, electrolyte oxidation or reduction cannot account for the capacity decay. Instead, the gradual decline is more likely associated with structural distortions of PB during repeated Ca^2+^ insertion, minor interfacial thickening on the HC surface, or slow accumulation of diffusion-related polarization in the host frameworks. These processes, rather than electrolyte degradation, are the primary contributors to the long-term capacity fading in Ca-ion full cells.

In parallel with the Ca(ClO_4_)_2_ system, the electrochemical behavior of Ca-ion full cells employing PB as the cathode and HC as the anode was evaluated using the Ca(PF_6_)_2_ electrolyte (Figure 10c,d). Galvanostatic cycling was conducted within the voltage window of 0–2 V. The Ca(PF_6_)_2_ full cell delivered an initial discharge capacity of approximately 38 mAh g^−1^, which was significantly lower than the 55 mAh g^−1^ obtained for the Ca(ClO_4_)_2_ system. Although the Coulombic efficiency remained relatively high (≈98%), the Ca(PF_6_)_2_ electrolyte exhibited accelerated capacity fading compared to Ca(ClO_4_)_2_. By the 100th cycle, the discharge capacity had fallen to 22 mAh g^−1^, corresponding to a 42.1% reduction relative to the initial capacity. Several mechanistic factors collectively account for the inferior electrochemical performance and accelerated degradation of the Ca(PF_6_)_2_-based full cells. First, the PF_6_^−^ anion exhibits intrinsically lower thermodynamic and electrochemical stability and undergoes gradual hydrolysis even in the presence of trace moisture, generating HF and other reactive species. These byproducts can damage the PB framework and promote the growth of a resistive, fluorine-rich interphase on the HC, thereby increasing cell polarization. Second, PF_6_^−^ is highly susceptible to photolytic and thermal decomposition during synthesis and handling. Partial conversion to POF_3_ and other fluoride-containing species that are difficult to eliminate entirely undermines the long-term electrolyte stability and accelerates capacity decay. Third, PF_6_^−^ forms a more strongly coordinated solvation sheath around Ca^2+^ in ACN relative to ClO_4_^−^, leading to sluggish Ca^2+^ desolvation kinetics at the electrode–electrolyte interface. The kinetic barrier exhibits overpotential and reduces the efficiency of Ca^+^ entry into PB. In PF_6_^−^-based electrolytes, the interfacial chemistry at the HC anode is less stable. Decomposition products create a thicker, more resistant surface layer, which further inhibits reversible Ca^2+^ storage. These chemical, kinetic, and interfacial instabilities explain why Ca(PF_6_)_2_ full cells have lower capacity, poorer activation, and quicker capacity fading compared to Ca(ClO_4_)_2_.

**Figure 11:**
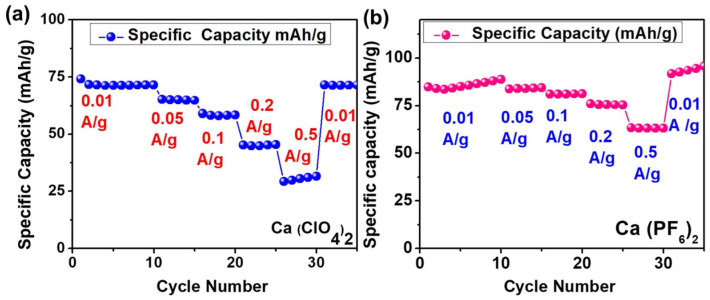
Rate capability of the PB-HC full cells with (**a**) 1 M Ca(ClO_4_)_2_ electrolyte in ACN and (**b**) 0.5 M Ca(PF_6_)_2_ electrolyte in ACN after drying with MS3A.

The PB-HC full cells were assessed in terms of their rate capability at various current densities (0.01, 0.05, 0.1, 0.2, and 0.5 A g^−1^). The discharge capacity of the Ca(ClO_4_)_2_ electrolyte dropped from 71.7 to 30.4 mAh g^−1^ as the current density increased. Recovering the current density to 0.01 A g^−1^ resulted in nearly full capacity recovery to 71.5 mAh g^−1^, with 99.72% retention compared with the initial low-rate cycling (Figure 11a). This recovery suggests that the capacity loss at higher rates is mostly caused by kinetic limits rather than irreversible structural degradation. In contrast, the Ca(PF_6_)_2_ electrolyte exhibited higher capacities of 85.7, 84.0, 81.1, 75.5, and 63.1 mAh g^−1^ across the same current range. It also displayed an activation-type rise in capacity when cycled again at 0.01 A g^−1^ (Figure 11b). This shows that Ca^2+^ transport and interfacial charge-transfer kinetics in the Ca(PF_6_)_2_ system gradually improve during initial cycling, potentially owing to stability or thinning of interphase layers on the PB or HC. Ca(ClO_4_)_2_ showed a more severe capacity decline with increasing current density than Ca(PF_6_). When comparing the capacities at cycles 26–30 to those achieved during the initial low-rate cycles, Ca(ClO_4_)_2_ showed a 57.6% loss, while Ca(PF_6_)_2_ showed only a 26.4% decline. Although Ca(ClO_4_)_2_ has stronger reversibility at low rates, the Ca(PF_6_)_2_ electrolyte provides improved rate tolerance owing to faster Ca^2+^ transport kinetics and more stable interface formation under high-rate circumstances.

The rate capability of PB‖HC full cells provides further insight into the effective Ca^2+^ transport characteristics of the electrolytes. As shown in Figure 11, the Ca(PF_6_)_2_–ACN electrolyte exhibits comparatively higher capacity retention at elevated current densities, which can be attributed to the formation of a more tightly coordinated Ca^2+^ solvation sheath that temporarily stabilizes charge transport under high polarization conditions. However, this strong ion association is accompanied by sluggish desolvation kinetics and increased interfacial resistance, ultimately leading to accelerated capacity decay during prolonged cycling. In contrast, the Ca(ClO_4_)_2_–ACN electrolyte shows a more pronounced capacity decrease with increasing current density but demonstrates nearly complete capacity recovery upon returning to low current operation (99.7% retention), indicating minimal irreversible degradation and superior long-term ion transport reversibility. This behavior reflects weaker Ca^2+^–anion interactions and reduced ion pairing, which facilitate more efficient desolvation and interfacial charge transfer. Collectively, these results highlight that while Ca(PF_6_)_2_ may support transient high-rate operation, Ca(ClO_4_)_2_ offers more favorable effective Ca^2+^ transport for sustained and reversible calcium-ion battery operation [5,6,33,34].

## 4. Conclusions

In this study, we thoroughly investigated the critical roles of electrolyte chemistry, solvent selection, and moisture management in enabling reversible and durable CIB functioning. Low-cost synthesis of Ca(ClO_4_)_2_ and Ca(PF_6_)_2_ salts using strict dehydration processes demonstrated that typically neglected trace quantities of water fundamentally control the Ca^+^ transport kinetics, interfacial stability, and long-term electrochemical performance. ACN was found to be the best solvent because of its high salt solubility, which allows for reversible Ca plating/stripping, whereas carbonate-based solvents cause fast polarization and cell failure. This study introduced MS3A-assisted dehydration, which successfully eliminated residual moisture beyond vacuum drying, reducing the water content to the ppm level and significantly stabilizing the electrolyte. This approach enabled PB half cells to produce high reversible capacities, whereas PB-HC full cells could run stably for 240 cycles with ≈99% Coulombic efficiency and an insignificant capacity fade when utilizing Ca(ClO_4_)_2_. Ca(PF_6_)_2_, despite having an excellent rate capability, showed accelerated capacity loss owing to inherent anion instability and interfacial deterioration. Overall, these results establish moisture-controlled electrolyte engineering as an essential design principle for multivalent batteries and offer a viable and scalable pathway for high-performance CIBs. The results revealed here are broadly applicable to various multivalent systems and offer a significant step toward sustainable, low-cost energy storage solutions beyond lithium.

## Figures and Tables

**Table 1:** EDX composition result for Ca(PF_6_)_2_.

Element	Wt%	Atomic %
O	14.51	21.04
F	43.87	53.56
P	11.52	8.63
Ca	28.32	16.39
Ag	1.77	0.38
Total	100.00	100.00

**Table 2:** Results of the Karl Fischer titration. All solutions used ACN as a solvent at a salt:solvent weight ratio of 5:95.

Sample	Result of Karl Fischer Titration (ppm)
Acetonitrile	10.3
Ca(ClO_4_)_2_ commercial	11,281.2
Ca(ClO_4_)_2_ commercial + MS3A	4560.1
Synthesized Ca(ClO_4_)_2_ vacuum dried	802.3
Synthesized Ca(ClO_4_)_2_ vacuum dried + MS3A	11.2
Synthesized Ca(PF_6_)_2_ + MS3A	10.3

## Data Availability

All materials, data, and associated protocols contained in this manuscript can be made available to readers upon request.

## References

[1] Placke T., Kloepsch R., Dühnen S., Winter M. (2017). Lithium ion, lithium metal, and alternative rechargeable battery technologies: The odyssey for high energy density. J. Solid State Electrochem..

[2] Tanaji Salunkhe T., Yoo J.H., Lee S.-W., Kim I.T. (2024). Exploring inexpensive electrodes for safer and evolved dual-ion batteries using modified electrolytes for enhanced energy density. J. Electroanal. Chem..

[3] Tanaji Salunkhe T., Bathula B., Rao Gurugubelli T., Pammi S.V.N., Yoo K. (2024). Highly efficient Z scheme heterojunction of colloidal SnO_2_ quantum dots grafted g-C3N4 for the degradation of rhodamine B under visible light. Results Phys..

[4] Liang Y., Dong H., Aurbach D., Yao Y. (2020). Current status and future directions of multivalent metal-ion batteries. Nat. Energy.

[5] Wei Q., Zhang L., Sun X., Liu T.L. (2022). Progress and prospects of electrolyte chemistry of calcium batteries. Chem. Sci..

[6] Taghavi-Kahagh A., Roghani-Mamaqani H., Salami-Kalajahi M. (2024). Powering the future: A comprehensive review on calcium-ion batteries. J. Energy Chem..

[7] Wang J., Yu R., Jiang Y., Qiao F., Liao X., Wang J., Huang M., Xiong F., Cui L., Dai Y. (2024). High-solvation electrolytes for ultra-stable calcium-ion storage. Energy Environ. Sci..

[8] Wang L., Riedel S., Zhao-Karger Z. (2024). Challenges and Progress in Anode-Electrolyte Interfaces for Rechargeable Divalent Metal Batteries. Adv. Energy Mater..

[9] Gummow R.J., Vamvounis G., Kannan M.B., He Y. (2018). Calcium-Ion Batteries: Current State-of-the-Art and Future Perspectives. Adv. Mater..

[10] Arroyo-de Dompablo M.E., Ponrouch A., Johansson P., Palacín M.R. (2020). Achievements, Challenges, and Prospects of Calcium Batteries. Chem. Rev..

[11] Salunkhe T.T., Kim I.T. (2024). Expanded Graphite as a Superior Anion Host Carrying High Output Voltage (4.62 V) and High Energy Density for Lithium Dual-Ion Batteries. Micromachines.

[12] Aurbach D., Skaletsky R., Gofer Y. (1991). The Electrochemical Behavior of Calcium Electrodes in a Few Organic Electrolytes. J. Electrochem. Soc..

[13] Hansel B., Burkmann K., Störr B., Pätzold C., Mertens F., Bertau M. (2025). Identification of Electrolyte Salts in Lithium-Ion Battery Black Mass. Chem. Ing. Tech..

[14] Chen M., Zhang J., Ji X., Fu J., Feng G. (2022). Progress on predicting the electrochemical stability window of electrolytes. Curr. Opin. Electrochem..

[15] Borodin O. (2019). Challenges with prediction of battery electrolyte electrochemical stability window and guiding the electrode–electrolyte stabilization. Curr. Opin. Electrochem..

[16] Lipson A.L., Pan B., Lapidus S.H., Liao C., Vaughey J.T., Ingram B.J. (2015). Rechargeable Ca-Ion Batteries: A New Energy Storage System. Chem. Mater..

[17] Xu Z.-L., Park J., Wang J., Moon H., Yoon G., Lim J., Ko Y.-J., Cho S.-P., Lee S.-Y., Kang K. (2021). A new high-voltage calcium intercalation host for ultra-stable and high-power calcium rechargeable batteries. Nat. Commun..

[18] Kjeldgaard S., Dugulan I., Mamakhel A., Wagemaker M., Iversen B.B., Bentien A. (2021). Strategies for synthesis of Prussian blue analogues. R. Soc. Open Sci..

[19] Tanaji Salunkhe T., Bathula B., Tae Kim I., Thirumal V., Yoo K. (2024). Synergistic integration of MoS_2_ nanopetals and SnO_2_ quantum dots for enhanced supercapacitor performance. J. Electroanal. Chem..

[20] Misra M., Srivastava A.K., Kadam A.N., Salunkhe T.T., Kumar V., Nikalje A.P.G. (2024). Substantial enhancement of optoelectronics and piezoelectric properties of novel hollow ZnO nanorods towards efficient flexible touch and bending sensor. Colloids Surf. A Physicochem. Eng. Asp..

[21] Williams D.B.G., Lawton M. (2010). Drying of Organic Solvents: Quantitative Evaluation of the Efficiency of Several Desiccants. J. Org. Chem..

[22] Majano G., Mintova S. (2010). Mineral oil regeneration using selective molecular sieves as sorbents. Chemosphere.

[23] Song H., Su J., Wang C. (2021). Multi-Ions Electrolyte Enabled High Performance Voltage Tailorable Room-Temperature Ca-Metal Batteries. Adv. Energy Mater..

[24] Dyson P., Tilmann G. (2005). Miscellaneous Reactions. Metal Catalysed Reactions in Ionic Liquids.

[25] Keyzer E.N., Matthews P.D., Liu Z., Bond A.D., Grey C.P., Wright D.S. (2017). Synthesis of Ca(PF_6_)_2_, formed via nitrosonium oxidation of calcium. Chem. Commun..

[26] Miyashita T., Yasuda K., Uda T. (2025). Kinetics and mechanism of hydrolysis of PF6− accelerated by H^+^ or Al^3+^ in aqueous solution. Environ. Sci. Water Res. Technol..

[27] Tyler J.L., Sacci R.L., Nanda J. (2021). Anion Coordination Improves High-Temperature Performance and Stability of NaPF6-Based Electrolytes for Supercapacitors. Energies.

[28] Melemed A.M., Khurram A., Gallant B.M. (2020). Current Understanding of Nonaqueous Electrolytes for Calcium-Based Batteries. Batter. Supercaps.

[29] Edge J.S., O’Kane S., Prosser R., Kirkaldy N.D., Patel A.N., Hales A., Ghosh A., Ai W., Chen J., Yang J. (2021). Lithium ion battery degradation: What you need to know. Phys. Chem. Chem. Phys..

[30] Hu C., Geng M., Yang H., Fan M., Sun Z., Yu R., Wei B. (2024). A Review of Capacity Fade Mechanism and Promotion Strategies for Lithium Iron Phosphate Batteries. Coatings.

[31] Jayawardana C., Rodrigo N., Parimalam B., Lucht B.L. (2021). Role of Electrolyte Oxidation and Difluorophosphoric Acid Generation in Crossover and Capacity Fade in Lithium Ion Batteries. ACS Energy Lett..

[32] Duy Anh C., Kim Y.J., Ngoc Vo T., Kim D., Hur J., Khani H., Kim I.T. (2023). Three-dimensional electrodes in hybrid electrolytes for high-loading and long-lasting calcium-ion batteries. Chem. Eng. J..

[33] Gao X., Liu X., Mariani A., Elia G.A., Lechner M., Streb C., Passerini S. (2020). Alkoxy-functionalized ionic liquid electrolytes: Understanding ionic coordination of calcium ion speciation for the rational design of calcium electrolytes. Energy Environ. Sci..

[34] Gerdroodbar A.E., Alihemmati H., Safavi-Mirmahaleh S.-A., Golshan M., Damircheli R., Eliseeva S.N., Salami-Kalajahi M. (2023). A review on ion transport pathways and coordination chemistry between ions and electrolytes in energy storage devices. J. Energy Storage.

